# Integrating intrapreneurial self-capital, cultural intelligence, and gender in Chinese international education: pathways to flourishing

**DOI:** 10.3389/fpsyg.2024.1358055

**Published:** 2024-01-29

**Authors:** Tianran Dong, Xuetao Li

**Affiliations:** College of Fashion and Design, Donghua University, Shanghai, China

**Keywords:** Chinese international students, cross-cultural adaptation, cultural intelligence, gender differences, intrapreneurial Self capital, psychological well-being

## Abstract

**Background:**

This study investigates the complex interplay between Intrapreneurial Self Capital, Cultural Intelligence, and gender, and their collective influence on the flourishing of Chinese international students in foreign academic settings. As global interconnectivity intensifies, the increasing number of Chinese students seeking education abroad presents a unique opportunity to examine the psychological and sociocultural dynamics of this demographic.

**Aim:**

Central to our investigation is the role of Cultural Intelligence, a crucial competency for navigating diverse environments, and Intrapreneurial Self Capital, a composite of psychological resources instrumental in educational and career success. The study also explores the mediating role of Cultural Intelligence in the relationship between Intrapreneurial Self Capital and student flourishing, and examines how gender moderates this dynamic.

**Method:**

The research engaged 508 Chinese international students, utilizing a variety of social networks for participant recruitment. The survey, conducted via Qualtrics, focused on a diverse range of students across different educational levels and disciplines. A moderated mediation model was tested to examine the mediation effect of cultural intelligence on the relationship between intrapreneurial self-capital and flourishing, with gender serving as a moderating variable.

**Results:**

Our findings reveal significant insights into how Intrapreneurial Self Capital and Cultural Intelligence contribute to the personal and professional development of Chinese international students. Overall, the results suggest that the impact of Intrapreneurial Self Capital on various cognitive qualities (Metacognitive Cultural Intelligence, Cognitive Cultural Intelligence, Motivational Cultural Intelligence and Behavioral Cultural Intelligence) is moderated by gender, highlighting the importance of considering gender differences in this context. Related to the prediction of Flourishing, the direct effect of Intrapreneurial Self Capital on flourishing is notably strong. However, the mediating roles of Metacognitive, Cognitive, and Behavioral aspects of Cultural Intelligence show different levels of influence.

**Implications:**

The study underscores the need for educational institutions to adopt holistic approaches in fostering student well-being and success, accounting for the nuanced effects of cultural and gender dynamics. These results have significant implications for the development of targeted educational programs and training, aimed at enhancing the international educational experience for students and professionals.

## Introduction

In an era marked by unprecedented global interconnectivity and cultural exchange, the landscape of international education, particularly within the context of Chinese students pursuing academic endeavors abroad, has become a fertile ground for exploring the interplay of various psychological and sociocultural factors (Wilczewski and Alon, 2023). The surge in Chinese international education is a phenomenon of immense importance. It reflects not only the global aspirations of Chinese students but also underscores a complex tapestry of opportunities and challenges that these students navigate (Kang and Hwang, 2022). This educational migration is more than a pursuit of academic excellence; it embodies a journey of personal and professional transformation, fraught with both unique opportunities and formidable challenges. As such, understanding these dynamics from an individual perspective is crucial (Zhu and O’Sullivan, 2022).

Central to this exploration is the concept of Cultural Intelligence, a multifaceted competency allowing individuals to thrive in culturally diverse environments (Van Dyne et al., 2016). Cultural Intelligence’s significance in the context of international education cannot be overstated, especially for students immersed in foreign academic and cultural settings (Van Dyne et al., 2015). This intelligence, which encompasses various dimensions, is a key factor in enhancing students’ adaptability and well-being, enabling them to navigate and flourish within these complex environments (Bai and Wang, 2022).

Another critical element is Intrapreneurial Self Capital, an emerging construct within educational psychology and career development. Encompassing a holistic blend of psychological resources, Intrapreneurial Self-capital has been identified as a vital contributor to educational success and career preparedness (Alessio et al., 2019). Its multifaceted nature, which includes aspects like resilience, creativity, and proactivity, positions Intrapreneurial Self-capital as an essential tool for students grappling with the demands of modern education and career landscapes (Guo et al., 2022).

Beyond academic performance, the concept of personal flourishing has gained traction, advocating for a more holistic understanding of student success. This approach recognizes that true success encompasses psychological, emotional, and social well-being, in addition to academic achievement. Thus, educational institutions are increasingly called upon to adopt a more comprehensive approach to student development, one that fosters personal flourishing in all its dimensions.

Our study also considers the mediating role of Cultural Intelligence in the relationship between Intrapreneurial Self-capital and international students’ flourishing. We posit that Cultural Intelligence could serve as a crucial link between the internal resources provided by Intrapreneurial Self-capital and the external challenges encountered in international educational settings. Furthermore, we explore the moderating role of gender in this dynamic, acknowledging the sparse research in this area and the need for a more nuanced understanding of how gender influences the development and application of Cultural Intelligence, particularly among Chinese international students.

In sum, our paper seeks to unravel the intricate relationship between Intrapreneurial Self Capital, Cultural Intelligence, and gender, and their collective impact on the flourishing of Chinese international students. By examining these interconnections, we aim to provide valuable insights that could inform the development of tailored educational and training programs, ultimately enhancing the international educational experience for students and professionals alike.

## Literature review

### Chinese international education: opportunities and challenges

The landscape of Chinese international education has evolved significantly, with an increasing number of students pursuing education abroad. This trend presents a unique set of opportunities and challenges, which are crucial to understand from an individual perspective (Xu and Shubo, 2023).

Educational migration offers Chinese students unique opportunities for personal and professional development. As found in the work of Bodycott and Lai (2012), studying abroad significantly enhances language proficiency, cultural understanding, and employability in the global job market. Furthermore, Wei (2013) highlights that international exposure broadens students’ worldviews, fostering critical thinking and adaptability.

However, this journey is not without its challenges (Chen et al., 2022). According to Spencer-Oatey and Xiong (2006), cultural adaptation remains a significant hurdle for many Chinese students, often leading to social isolation and academic challenges. This cultural adjustment is compounded by the language barrier, as identified by Sherry et al. (2010), which can impede both academic performance and social integration.

The mismatch between the educational experiences abroad and the expectations within the Chinese job market can affect career trajectories of returning students (Guo et al., 2020).

The literature reveals a complex picture of opportunities and challenges faced by Chinese international students. The benefits of personal growth and enhanced employability coexist with the difficulties of cultural adaptation and educational recognition. This understanding is vital for stakeholders in international education to develop support systems that cater to the needs of these students.

### Cultural intelligence: how to function effectively in front of cultural differences

Cultural Intelligence (hereafter, CQ) is a multifaceted competency that allows individuals to function effectively in culturally diverse settings. Earley and Ang (2003) define it as the capability to relate and work effectively across cultures. This concept has gained prominence in the context of globalization, where cross-cultural interactions are commonplace.

Cultural Intelligence is composed of four distinct dimensions, being the first the Metacognitive CQ: This dimension refers to the level of conscious cultural awareness during cross-cultural interactions. It involves high-level cognitive processes, such as cultural mindfulness and reflective thinking about one’s own and others’ cultural knowledge. Thomas (2008) describe metacognitive CQ as the ability to be aware of and understand the cultural contexts of both oneself and others. The second, cognitive CQ, pertains to the knowledge of norms, practices, and conventions in different cultural settings. Cognitive CQ includes understanding the similarities and differences across cultures, which encompasses knowledge of the economic, legal, and social structures of different countries.

The third is the motivational CQ. This dimension involves the drive and interest to adapt and function effectively in culturally diverse situations. It is characterized by an intrinsic interest in intercultural experiences and a confidence in dealing with them. As per Van Dyne, Ang, and Livermore, motivational CQ fuels an individual’s ability to direct attention and energy toward learning about and functioning in situations characterized by cultural diversity (Van Dyne et al., 2010). Finally, the behavioral CQ: The ability to exhibit appropriate actions when interacting with people from different cultures. It involves having a range of flexible verbal and non-verbal behaviors. Spencer-Oatey and Franklin (2012) discuss how behavioral CQ enables individuals to adapt their behaviors to different cultural contexts.

Recent studies have linked cultural intelligence to improved wellbeing among students, particularly those studying in international or culturally diverse environments. A study by Ott and Michailova (2018) found that students with high levels of cultural intelligence, especially in the motivational and behavioral dimensions, exhibit better adaptation skills in new cultural settings, which correlates with higher levels of overall wellbeing. Furthermore, further research indicates that students with high metacognitive and cognitive CQ are less likely to experience cultural shock, leading to a more positive experience in foreign academic environments (Goh et al., 2021).

In conclusion, cultural intelligence significantly influences the ability of students to navigate and thrive in culturally diverse academic settings. Each dimension of Cultural Intelligence plays a crucial role in enhancing students’ adaptability and wellbeing, underlining the importance of developing these skills in global education contexts.

### Intrapreneurial self capital: key resource for educational success

Intrapreneurial Self Capital (hereafter, ISC) is an emerging construct in the field of educational psychology and career development, representing a holistic blend of psychological resources (Di Fabio and Kenny, 2018). Di Fabio and colleagues offer a comprehensive view of ISC, describing it as a higher-order construct comprising seven specific constructs: core self-evaluation, resilience, creative self-efficacy, grit, goal mastery, determination, and attentiveness (Di Fabio, 2014). This multifaceted nature underscores its importance in navigating complex educational environments and adapting to dynamic academic and career landscapes. The components of Intrapreneurial Self Capital, as outlined by Di Fabio and colleagues, is a high order construct that include seven sub-dimensions: Core Self-Evaluation: This aspect reflects an individual’s fundamental appraisal of their own abilities and worth. Resilience: this involves the capacity to recover quickly from challenges and adapt to change. Resilience in the context of ISC involves the ability to bounce back from setbacks and adapt to changing circumstances. As per Luthans and colleagues, it’s a vital component in maintaining positive educational outcomes in the face of adversity (Luthans et al., 2006). Creative Self-Efficacy: This refers to the belief in one’s ability to produce creative outcomes. Creativity in ISC is not just about novel ideas but also the application of these ideas in problem-solving. Amabile (1997) emphasizes the role of creativity in enhancing personal and professional effectiveness in various settings, including education. Grit: Defined as perseverance and passion for long-term goals, contributing to sustained effort and interest over years despite failure, adversity, and plateaus in progress. Goal Mastery: The ability to effectively set and achieve personal and academic goals. This aspect focuses on the setting and achieving of personal and academic goals. Locke and Latham (2002) discuss how goal-setting theory plays a significant role in personal development and success. Determination: A quality that entails a strong commitment to achieve despite obstacles and setbacks. Attentiveness: The ability to maintain focus and be vigilant in one’s pursuits.

The validity of the construct and its psychometric properties have been empirically explored in western (McIlveen and Di Fabio, 2018; Palazzeschi et al., 2019; Puigmitja et al., 2019) and eastern countries (Bee Seok et al., 2019, 2020; Malekiha, 2020), showing its cross-cultural significance and relevance for the academic contexts.

The study of Intrapreneurial Self-capital’s impact on educational success is gaining momentum. McIlveen and Di Fabio, (2018) suggest that students with higher Intrapreneurial Self-capital levels exhibit better adaptability to academic challenges and engagement in studies. They also note the relevance of Intrapreneurial Self-capital in career readiness, emphasizing its importance in transitioning from education to the workforce (Di Fabio, 2021; Rosen and Di Fabio, 2023).

A study found a significant correlation between Intrapreneurial Self-capital and academic achievement, indicating that the diverse components of Intrapreneurial Self-capital, such as resilience and determination, contribute to better academic outcomes, underscoring its value for students preparing for professional life (Di Fabio et al., 2017).

In conclusion, Intrapreneurial Self Capital is a key resource in educational contexts, contributing significantly to academic success and career preparedness (Singh, 2021; Gutiérrez-Carrasco et al., 2022; López-Núñez et al., 2022). Its multifaceted nature, encompassing elements like proactivity, resilience, and creativity, makes it an essential construct for students navigating the complex demands of modern education and career landscapes (Henter and Nastasa, 2021; Iliashenko et al., 2023).

### More than academic performance: students’ personal flourishing

In the contemporary educational discourse, there has been a paradigm shift toward a more holistic understanding of students’ success. Traditionally, academic performance, measured through grades, test scores, and degree attainment, has been the primary indicator of success. However, this narrow focus overlooks the multifaceted nature of student development and well-being. The concept of personal flourishing comes into play as a more comprehensive measure, encompassing not only academic achievement but also psychological, emotional, and social well-being (Seligman and Csikszentmihalyi, 2000).

Personal flourishing refers to a state where individuals experience a high level of well-being and life satisfaction. This concept, rooted in positive psychology, extends beyond the absence of mental health issues to include the presence of positive emotions, engagement, relationships, meaning, and accomplishment (Seligman, 2011). In the context of education, personal flourishing is characterized by students experiencing growth in various dimensions of their life, not limited to academic achievements (Gan and Cheng, 2021).

While academic performance is a significant aspect of student life, it is not the sole determinant of personal flourishing. The interplay of mental health and academic performance is also critical. Studies have demonstrated that higher levels of mental health, including lower instances of anxiety and depression, are associated with better academic outcomes (Stallman, 2010). This relationship underlines the importance of addressing mental health as part of comprehensive educational success strategies. Flourishing students are those who engage with their studies in a manner that promotes their overall well-being. This includes developing resilience, fostering a growth mindset, and maintaining a healthy balance between academic and personal life.

Psychological and emotional well-being are critical components of personal flourishing. Students who are flourishing demonstrate resilience, coping effectively with stress and setbacks. They also exhibit higher levels of self-efficacy and self-esteem, which contribute to their overall sense of well-being (Bandura et al., 1999). This theory has been substantiated by studies showing a strong correlation between self-efficacy and academic achievement (Pajares, 1996; Zimmerman, 2000).

Social relationships and community engagement are also integral to personal flourishing. Positive relationships with peers, faculty, and the wider community can enhance students’ sense of belonging and purpose, thereby contributing to their overall well-being (Putnam, 2000). Active involvement in extracurricular activities and community service can further foster a sense of connection and accomplishment.

In conclusion, redefining student success to include personal flourishing represents a more inclusive and holistic approach to education (Di Fabio et al., 2017). By focusing on the overall well-being of students, educational institutions can contribute to the development of individuals who are not only academically proficient but also psychologically robust, emotionally balanced, and socially engaged, equipping them with the necessary tools to thrive in all aspects of their lives.

### Mediating role of cultural intelligence in the relationships between ISC and international students’ flourishing

The predictive value of Intrapreneurial Self Capital in the context of international students’ academic and career success is an area of growing interest. Intrapreneurial Self Capital, with its components such as resilience, creative self-efficacy, and determination, appears to play a crucial role in the success of these students who face unique challenges in a foreign educational environment (Palazzeschi et al., 2019). In particular, the attributes of Intrapreneurial Self Capital can be instrumental in helping international students navigate cultural, linguistic, and academic barriers, thereby enhancing their overall educational experience and personal flourishing. As some studies showed, self-efficacy, a key component of Intrapreneurial Self-capital, emerges as a prominent factor in explaining the Cultural Intelligence (MacNab and Worthley, 2012).

Moreover, the relationship between Intrapreneurial Self Capital and international students’ success may be further elucidated through the mediating role of Cultural Intelligence. Cultural Intelligence could serve as a bridge linking the internal resources of Intrapreneurial Self Capital with the external challenges faced in international education settings. For instance, a high level of resilience and goal mastery (components of Intrapreneurial Self Capital) might enable a student to adapt more effectively to a new cultural environment, a process potentially mediated by their level of Cultural Intelligence. This suggests that while Intrapreneurial Self Capital equips students with internal psychological resources, Cultural Intelligence enables them to apply these resources effectively in culturally diverse settings (Dolce and Ghislieri, 2022).

Empirical research is needed to explore this potential mediating role of Cultural Intelligence in the relationship between Intrapreneurial Self Capital and the success of international students. Such research could provide valuable insights into how educational institutions and policymakers can better support international students by not only fostering Intrapreneurial Self Capital but also enhancing their Cultural Intelligence, thereby ensuring a more holistic approach to international education.

This perspective opens up new avenues for understanding the multifaceted challenges faced by international students and highlights the importance of an integrated approach that considers both personal attributes (like Intrapreneurial Self Capital) and the ability to navigate cultural differences (through Cultural Intelligence) in ensuring their academic and career success in international contexts.

### The moderating role of gender in the relationship between intrapreneurial self-capital and cultural intelligence among Chinese international students

The investigation into the moderating effects of gender on the relationship between Intrapreneurial Self Capital and Cultural Intelligence in Chinese international students offers a novel perspective in understanding how these elements interact within a cross-cultural context. This analysis is particularly relevant given the sparse research addressing the direct impact of gender differences on Cultural Intelligence levels and their subsequent influence on career success and cultural adaptability.

Research to date has frequently treated gender as a control variable rather than a focal point of study. For instance, Aslam et al. (2016) and Jyoti and Kour (2017) included gender as a demographic variable in their studies but did not delve deeply into its specific impacts. In a study involving 335 global managers in India, Jyoti and Kour (2017) found that gender had an insignificant impact on the primary variables of Cultural Intelligence, job performance, and cross-cultural adjustments. This finding suggests that the influence of gender on Cultural Intelligence and related outcomes may be more nuanced than previously assumed.

However, there are indications that gender differences do exist in specific dimensions of Cultural Intelligence. Zhou and Charoensukmongkol (2022) noted that there is a moderating role of gender in the effectiveness of cultural intelligence on customer qualifications skills. Conversely, (Khodadady and Ghahari (2012) observed that females exhibited higher levels of metacognitive CQ, suggesting a greater propensity for reflective and adaptive thinking in unfamiliar cultural settings (Khodadady and Ghahari, 2012).

An interesting dimension to this discourse is presented by Mandell and Pherwani (2003), who found that females generally exhibited higher emotional intelligence than males. This aspect of emotional intelligence could potentially contribute to higher motivational scores in Cultural Intelligence, as motivation can be partially driven by emotional responses to environmental stimuli. The ability to harness emotions effectively may offer females an advantage in adapting motivationally in diverse cultural contexts.

Given these disparate findings, there is a clear need for more focused research examining the relationship between Cultural Intelligence and gender, particularly among specific groups such as Chinese international students. Such studies would contribute significantly to our understanding of how gender influences the development and application of Cultural Intelligence in multicultural environments.

In conclusion, while existing literature provides some insights into the complex relationship between gender, Intrapreneurial Self Capital and Cultural Intelligence, it also highlights a significant gap in our understanding. More nuanced and focused research is required to unpack the layers of this relationship, particularly in the context of Chinese international students, who navigate unique cultural and educational landscapes. The exploration of how Intrapreneurial Self Capital, as a composite of personal and professional skills, interacts with gender to influence Cultural Intelligence could provide valuable insights into tailoring educational and training programs for international students and professionals.

**Figure 1:**
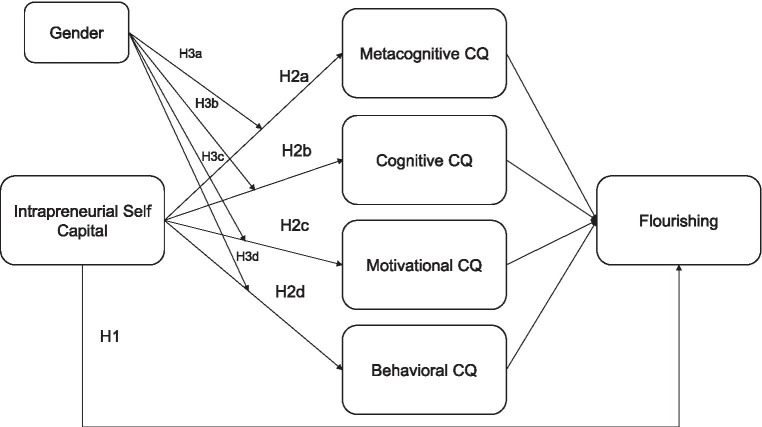
Research model.

Following the previously revised evidence, the present study proposes this research model displayed in Figure 1.

The following hypotheses are proposed:

*H1*: Intrapreneurial Self-capital will predict Flourishing among Chinese international students.

*H2*: Cultural intelligence will mediate the relationship between Intrapreneurial Self-capital and Flourishing among Chinese international students.

In more detail:

*H2a*: Metacognitive CQ will mediate the relationship between Intrapreneurial Self-capital and Flourishing among Chinese international students.

*H2b*: Cognitive CQ will mediate the relationship between Intrapreneurial Self-capital and Flourishing among Chinese international students.

*H2c*: Motivational CQ will mediate the relationship between Intrapreneurial Self-capital and Flourishing among Chinese international students.

*H2d*: Behavioral CQ will mediate the relationship between Intrapreneurial Self-capital and Flourishing among Chinese international students.

*H3*: Chinese international students’ gender will moderate the relationship between Intrapreneurial Self-capital and Cultural intelligence.

In more detail:

*H3a*: Chinese international students’ gender will moderate the relationship between Intrapreneurial Self-capital and Metacognitive CQ.

*H3b*: Chinese international students’ gender will moderate the relationship between Intrapreneurial Self-capital and Cognitive CQ.

*H3c*: Chinese international students’ gender will moderate the relationship between Intrapreneurial Self-capital and Motivational CQ.

*H3d*: Chinese international students’ gender will moderate the relationship between Intrapreneurial Self-capital and Behavioral CQ.

## Method

### Participants, procedure, and statistical analyses

This study involved a sample of 508 Chinese international students. These participants were contacted through various social networks, which provided a convenient and efficient means of reaching a broad and diverse group within this demographic (WeChat, Sina Weibo, QQ, Douyin, Zhihu, Linkedin, and Renren). The selection of these platforms was based on their prevalence and usage patterns among Chinese international students. Participants were 103 males (20.8%), (Mean age = 23.23 years; S.D. = 4.80), 57.8% were enrolled in Senior secondary education while 30.5% were enrolled in Master’s and 21.7% in Ph.D. programs. Regarding the discipline of the studies, 8.1% were in health-related studies, 7.9% education, 39.7% in engineering, 35.2% in banking, finances, and economic studies, 6.5% in technologies, 2.5% commerce and tourism.

Invitations to participate in the study were disseminated through these networks, outlining the purpose of the research and assuring confidentiality and anonymity. The survey was administered using Qualtrics, a robust online survey platform known for its user-friendly interface and advanced data collection capabilities. Participants were given a unique link to access the survey, which remained open for a period of 4 weeks, between March and April 2023. During this time, two reminders were sent out via the same social networks to encourage participation and ensure a comprehensive response rate. The Ethical Committee of the Donghua University provided approval for the present study. Prior to participation, all respondents were required to give their informed consent, which was facilitated through an electronic form on the Qualtrics platform. This form detailed the purpose of the study, the voluntary nature of participation, and the measures in place to safeguard participant privacy and data confidentiality. No personal data have been collected, more than the start date and finish date and percentage of completion of the survey. Those participants that do not reach the 100% of survey completion have been eliminated (response rate 80.2%). Data have been examined with SPSS version 25 software and Jamovi version 2.4. (Jamovi, 2023) for correlational analyses and Confirmatory Factor Analyses. PROCESS macros for SPSS (Hayes) Model 7 was used for the moderated mediation model.

### Instruments

#### Intrapreneurial self capital

This variable was assessed with the Intrapreneurial Self Capital Scale (Di Fabio, 2014), including 28 items measured via a 5-point Likert scale (ranging from 1 = strongly disagree to 5 = strongly agree). some item examples are “Sometimes when I fail, I feel worthless” (Core Self-Evaluation), “Planning in advance can help avoid most future problems” (Grit), “I’m able to solve problems creatively” (Creative Self-Efficacy), “I’m able to achieve objectives despite obstacles” (Resilience), “One of my goals in training is to learn as much as I can” (Goal Mastery), “It’s simple for me to decide” (Determination), “When I must to take a decision, I like to stop and consider all possible options” (Attentiveness). Cronbach’s alpha coefficient in previous studies was 0.84 (Di Fabio, 2014), while in the present study is: 0.89 for the global scale. The McDonalds’ omega coefficient for the global scale is 0.97. The omegas’ values for the factors ranged from 0.67 for Resilience to 0.88 for Core Self-evaluation. Cronbach’s Alphas for the separate factors ranged from 0.65 for Resilience to 0.85 for Goal Mastery. Average Extracted Variance for the nine factors ranged 0.40 to 0.67. Adaptations of this scale in non-western societies, as Malaysia and Iran, have demonstrated adequate psychometric properties, indicating its potential cross-cultural applicability and relevance (Bee Seok et al., 2019, 2020; Malekiha, 2020).

#### Cultural intelligence

The Cultural Intelligence Scale (CQ) used in this study was developed by Van Dyne et al. (2015) in the Chinese version published by Schlägel and Sarstedt (2016). The CQS has 20 items divided into four dimensions. This scale includes four items for metacognitive CQ, six items for cognitive CQ, five items for motivational CQ and five items for behavioral CQ. All items were rated on a 7-point Likert scale (1 = strongly disagree, 7 = strongly agree). The reliability of the scale in the present study was found to be adequate (Cronbach’s Alpha = 0.91; Mc Donald’s omega = 0.94). The omega coefficient for the CQ factors ranged from 0.78 for Metacognitive CQ to 0.88 for the Cognitive CQ. The Average Extracted Variance for the factors ranged from 0.50 for Metacognitive CQ to 0.57 for the Behavioral CQ.

#### Flourishing

The Flourishing Scale Spanish version (FS-SV) (Ramírez-Maestre et al., 2017) of Diener et al.’s (2010) Flourishing Scale was used. This scale measures critical aspects of psychosocial functioning through eight items which provide a single well-being score. The instrument showed a reliability value of 0.89 in previous studies, while the reliability of the scale in the present study was found to be adequate (0.88). Examples of items: “My social relationships are supportive and rewarding,” “I lead a purposeful and meaningful life,” “I am optimistic about my future.”

Descriptive data as gender, age, educational qualification, discipline of studies and number of countries lived in were collected as categorical or continuous variables (age as number of years). Based on the recommendations from recent studies, Harman’s single factor test is no longer relevant (Hulland et al., 2018), but we try to apply procedural remedies to avoid Common method bias using a web-based survey which displays the scales for the different variables along different web-pages (Memon et al., 2023). By this procedure we avoid the location of the items in close proximity to one another.

## Results

### Confirmatory factor analysis

For the Cultural Intelligence scale, there is some controversial evidence about its structural invariance (Lin et al., 2012; Schlägel and Sarstedt, 2016). Hence, we conducted a Confirmatory Factor Analysis that reveals significant findings across its four dimensions. Each dimension’s indicators show strong statistical significance (*p* < 0.001) with varying standard estimates, indicating a robust association with their respective constructs. Metacognitive CQ 2 and 3, Cognitive CQ 7, 8, and 10, Motivational CQ 12 and 13, and Behavioral CQ 19 are particularly notable for their high standard estimates.

The overall model fit is assessed through several fit indices. The Chi-square test (χ^2^ = 798, df = 164, *p* < 0.001) suggests a significant discrepancy between the observed and expected covariance matrices, which is common in large samples. The RMSEA (Root Mean Square Error of Approximation) value of 0.0500 (90% CI: 0.0881–0.0820) indicates an acceptable model fit, falling within the commonly accepted threshold. The CFI (Comparative Fit Index) and TLI (Tucker-Lewis Index) values of 0.885 and 0.867, respectively, though slightly below the preferred threshold of 0.90, still suggest a reasonable fit. The SRMR (Standardized Root Mean Square Residual) value of 0.0943, being close to 0.08, also supports an acceptable model fit.

Overall, while the fit indices indicate that the model could benefit from further refinement, the strong indicator relationships suggest that the Cultural Intelligence Scale has substantial construct validity in its current form. Confirmatory Factor Analysis and the factor loadings are displayed in Table 1.

**Table 1:** Factor loadings for the Cultural Intelligence Scale.

Factor	Indicator	*Z*	*p*	Standardized factor loading
Metacognitive CQ	Metacognitive CQ 1	15.6	< 0.001	0.664
	Metacognitive CQ 2	21.2	< 0.001	0.827
	Metacognitive CQ 3	21.6	< 0.001	0.840
	Metacognitive CQ 4	11.7	< 0.001	0.523
Cognitive CQ	Cognitive CQ 5	16.3	< 0.001	0.675
	Cognitive CQ 6	15.3	< 0.001	0.643
	Cognitive CQ 7	21.2	< 0.001	0.816
	Cognitive CQ 8	21.5	< 0.001	0.821
	Cognitive CQ 9	16.9	< 0.001	0.694
	Cognitive CQ 10	20.3	< 0.001	0.792
Motivational CQ	Motivational CQ 11	20.3	< 0.001	0.793
	Motivational CQ 12	20.5	< 0.001	0.800
	Motivational CQ 13	20.0	< 0.001	0.783
	Motivational CQ 14	19.1	< 0.001	0.764
	Motivational CQ 15	14.8	< 0.001	0.632
Behavioral CQ	Behavioral CQ 16	17.8	< 0.001	0.721
	Behavioral CQ 17	20.9	< 0.001	0.808
	Behavioral CQ 18	20.7	< 0.001	0.799
	Behavioral CQ 19	22.3	< 0.001	0.845
	Behavioral CQ 20	14.7	< 0.001	0.623

### Descriptive statistics and Pearson’s correlation matrix

Intrapreneurial Self-Capital is moderately correlated with Motivational and Behavioral Cultural Intelligence, and less so with Metacognitive and Cognitive Cultural Intelligence (CQ), as Table 2 illustrates. There is a strong correlation between Intrapreneurial Self-Capital and Flourishing, suggesting a significant link. Metacognitive CQ correlates strongly with Behavioral CQ and moderately with Motivational CQ and Flourishing. The correlation between Metacognitive and Cognitive CQ is weaker, suggesting a more complex relationship. Cognitive CQ has moderate correlations with Motivational and Behavioral CQ, but a weaker correlation with Flourishing, indicating a less direct impact on well-being. Motivational CQ and Behavioral CQ are strongly related, highlighting their close connection in Cultural Intelligence. Motivational CQ also shows a notable correlation with Flourishing. Behavioral CQ’s moderate correlation with Flourishing emphasizes the role of adaptive behavior in cross-cultural well-being. Overall, these correlations offer a detailed view of the interplay between different personal and cognitive attributes.

**Table 2:** Descriptive statistics and Pearson’s’ correlation matrix (*N* = 508).

	*M*	S.D.	1	2	3	4	5
1. Intrapreneurial Self-Capital	3.72	0.551	–				
2. Metacognitive CQ	4.15	0.76	0.300***	–			
3. Cognitive CQ	2.85	0.91	0.203***	0.251***	–		
4. Motivational CQ	3.70	0.84	0.486***	0.431***	0.463***	–	
5. Behavioral CQ	4.03	0.76	0.395***	0.577***	0.387***	0.523***	–
6. Flourishing	4.14	0.68	0.760***	0.312***	0.200***	0.371***	0.415***

### Moderated mediation analyses

The results from the Model 7 involve Flourishing as the outcome variable, Intrapreneurial Self Capital as the independent variable, four mediators (Metacognitive CQ, Cognitive CQ, Motivational CQ, Behavioral CQ), and Gender as the moderating variable, with a sample size of 498.

**Figure 2:**
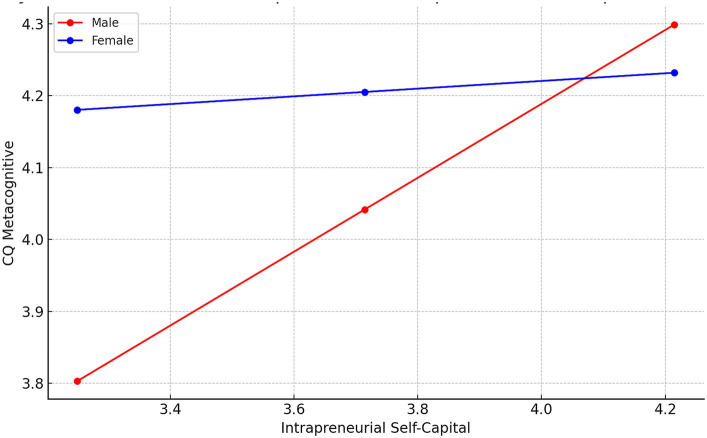
Moderation by Gender of the relationships between Intrapreneurial Self-capital and Metacognitive CQ.

First, being the outcome Metacognitive CQ, the model summary shows a moderate R-squared value of 0.1922. The coefficients for Intrapreneurial Self Capital and Gender are significant, as is their interaction term. This interaction indicates that the effect of Intrapreneurial Self Capital on Metacognitive CQ varies depending on the students’ gender. For the conditional effects of Intrapreneurial Self Capital at different gender groups, we see a significant effect for males, but not for females. The moderation graph is provided in Figure 2.

**Figure 3:**
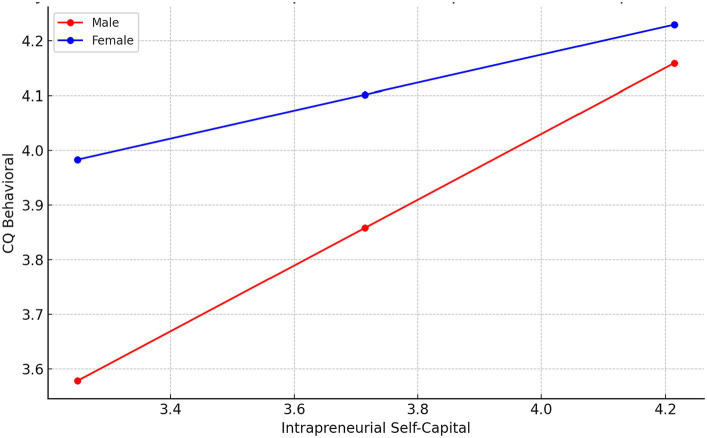
Moderation by Gender of the relationships between Intrapreneurial Self-capital and Cognitive CQ.

Moving on to Cognitive CQ, the model again shows a significant overall fit with an R-squared value of 0.0733. Similar to Cognitive CQ, the interaction between Intrapreneurial Self Capital and Gender is significant. The conditional effects reveal that Intrapreneurial Self Capital significantly predicts Cognitive CQ for males, but not for women, indicating a gender-specific effect (Figure 3).

**Figure 4:**
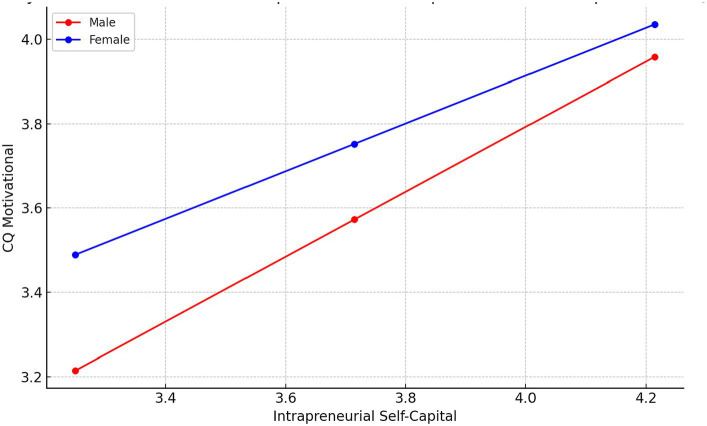
Moderation by Gender of the relationships between Intrapreneurial Self-capital and Motivational CQ.

Lastly, for Motivational CQ, the model demonstrates a stronger relationship with an R-squared value of 0.2617. The interaction between Intrapreneurial Self Capital and Gender is again significant, with Intrapreneurial Self Capital being a significant predictor of Motivational CQ for both genders, although the effect size varies. The Moderation graph for this relationships is displayed in Figure 4.

Overall, these results suggest that the impact of Intrapreneurial Self Capital on various cognitive qualities (Metacognitive CQ, Cognitive CQ, Motivational CQ) is moderated by gender, highlighting the importance of considering gender differences in this context. The significant interaction effects across different outcome variables underscore the nuanced relationship between the independent variable and mediators, shaped by the moderating influence of gender.

Regarding the Behavioral CQ, the model summary indicates a reasonably strong relationship, with an R-squared value of 0.2334. In the model, the constant is not significant, which is typical as it represents the expected value of Behavioral CQ when all other variables are zero. The coefficient for Intrapreneurial Self Capital is significant, indicating a strong positive relationship between Intrapreneurial Self Capital and Behavioral CQ.

**Figure 5:**
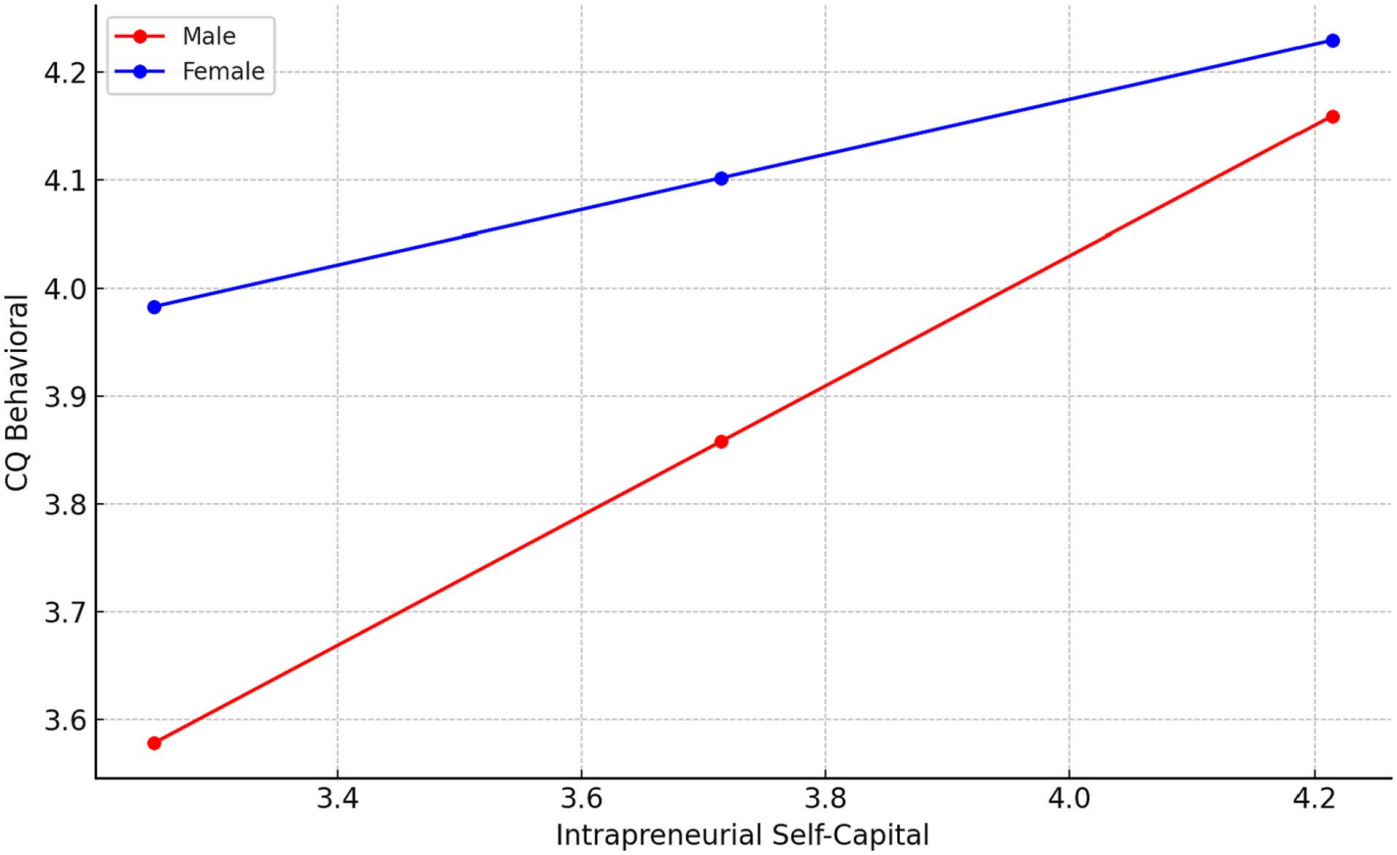
Moderation by Gender of the relationships between Intrapreneurial Self-capital and Behavioral CQ.

The coefficient for Gender is also significant and positive. The interaction term, is significant and negative. In the analysis of conditional effects, we see that for males the effect of Intrapreneurial Self Capital on Behavioral CQ is positive and strong. However, for females the effect remains significant but is weaker. This indicates that Intrapreneurial Self Capital is a more potent predictor of behavioral cultural intelligence among males compared to females. The Moderation graph for this relationships is displayed in Figure 5.

Finally, related to the prediction of Flourishing, the R-squared value is 59.87%.

The direct effect of Intrapreneurial Self Capital on flourishing is strong. However, the mediating roles of Metacognitive, Cognitive, and Behavioral aspects of Cultural Intelligence show different levels of influence. The Metacognitive CQ demonstrates minimal and statistically insignificant mediating effects for both genders. The index of moderated mediation is −0.0191 (95% CI [−0.0507; 0.0166]), indicating that this moderating effect of gender might not be statistically significant. Similarly, for the cognitive CQ, the index of moderated mediation is −0.0077 (95% CI [−0.0253; 0.0092]), suggesting that this moderating effect of gender is not statistically significant.

**Figure 6:**
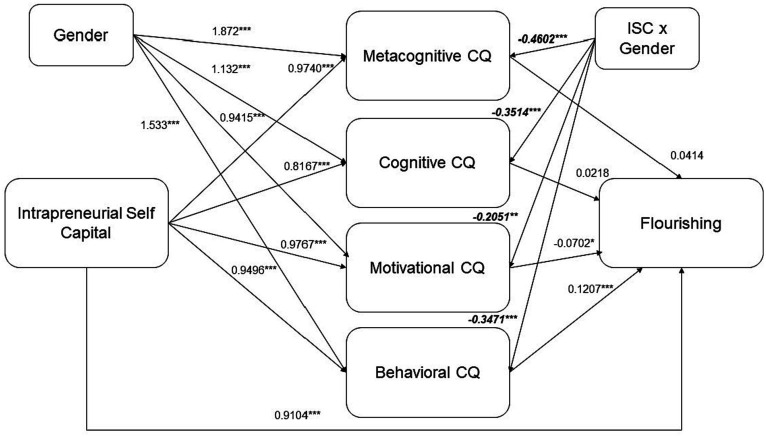
Unstandardized estimates for Model 7. *p < 0.05; **p < 0.01; ***p < 0.001, Values in italics for interaction terms.

In contrast, the Motivational CQ and Behavioral CQ exhibit more pronounced effects. The significant indirect effect via Motivational CQ, though small, indicates that motivational factors in cultural intelligence may contribute to how Intrapreneurial Self Capital impacts flourishing. The index for the motivational aspect is 0.0144 (95% CI [−0.0002; 0.0403]). This could imply that motivational factors in cultural intelligence might have a slightly more pronounced role in mediating the ISC-flourishing link for one gender over the other. Similarly, the Behavioral aspect shows a more substantial effect, especially for males. The index of moderated mediation is −0.0419 (95% CI [−0.0719; −0.0125]) suggests that this gender moderation is statistically significant. This finding is particularly noteworthy as it demonstrates a clear gender difference in how behavioral aspects of cultural intelligence mediate the relationship between ISC and flourishing (Figure 6).

## Discussion

This study aimed to explore the relationships among Intrapreneurial Self-capital, Cultural Intelligence, and Flourishing among Chinese international students, while also examining the moderating role of gender. The findings present a complex interplay between these factors, revealing significant insights into the dynamics of intrapreneurial capabilities, cultural adaptability, and well-being in a cross-cultural context.

The strong direct effect of Intrapreneurial Self-capital on flourishing underscores the importance of intrapreneurial qualities in enhancing well-being. This finding, supporting Hypothesis 1, aligns with existing literature emphasizing the role of self-initiative, resourcefulness, and resilience in promoting psychological health and adaptability, especially in cross-cultural environments. The substantial influence of Intrapreneurial Self-capital on flourishing indicates that fostering intrapreneurial skills may be crucial for the well-being of international students. This finding is in line with previous research showing the role of students’ Intrapreneurial Self-capital in predicting flourishing, academic performance and life satisfaction in other Eastern countries, as Malaysia (Bee Seok et al., 2020).

The mediating roles of the four dimensions of Cultural Intelligence (Metacognitive, Cognitive, Motivational, and Behavioral) presented a complex picture. Firstly, metacognitive and Cognitive CQ: Both showed minimal and statistically insignificant mediation effects. This suggests that the reflective thinking and knowledge aspects of cultural intelligence might not significantly influence how Intrapreneurial Self-capital translates into flourishing. These findings indicate that while these cognitive aspects are essential components of cultural intelligence, they may not be the primary mechanisms through which intrapreneurial self-capital contributes to students’ well-being. This research is according to other findings about the role of emotional constructs in explaining the impact of Intrapreneurial Self-capital on outcomes (Di Fabio and Saklofske, 2019).

Then, motivational and Behavioral CQ, in contrast, demonstrated more substantial mediating effects. Particularly, the Behavioral aspect of Cultural Intelligence, especially among males, played a significant role in mediating the relationship between Intrapreneurial Self-capital and flourishing. This implies that the ability to adapt behaviorally in a culturally diverse environment is a crucial factor in leveraging intrapreneurial skills for enhancing well-being. The motivational aspect, though smaller in effect, still indicates the importance of motivation in cultural adaptation as a pathway linking ISC to flourishing. These findings partially support Hypothesis 2, highlighting the nuanced roles different aspects of Cultural Intelligence play in this context. Our findings are aligned with other studies that showed the impact of Cultural Intelligence beyond the educative context on workers’ burnout when interacting with immigrants, highlighting the relevance of cultural competence in professional success (Puzzo et al., 2023).

The study’s exploration into the moderating effect of gender revealed significant insights. The impact of Intrapreneurial Self-capital on various dimensions of Cultural Intelligence was found to be moderated by gender. Specifically, Behavioral and Motivational CQ showed a more pronounced gender difference in their mediating effects. For instance, Behavioral CQ’s role as a mediator was stronger for males, suggesting that gender plays a crucial role in how behavioral adaptation in a new cultural environment influences the relationship between ISC and flourishing. These findings suggest that gender differences should be considered in understanding how intrapreneurial capabilities interact with cultural intelligence to impact well-being. The significant moderation by gender, especially in the Behavioral and Motivational dimensions of Cultural Intelligence, underscores the need for gender-sensitive approaches in facilitating the flourishing of international students. This supports Hypothesis 3 and its sub-components, highlighting the importance of considering gender in the dynamic interplay between intrapreneurial self-capital and cultural intelligence. This finding is in line with previous studies that have stated gender differences on the Cultural Intelligence-related outcomes (Davis, 2013; Mæland and Wattenberg, 2017). Hereafter, we need to recognize that other relevant variables, not taken into account in the present research, could be considered. Among the predictors of Cultural Intelligence, some studies have considered personality traits and its relationships with cultural adaptation via Cultural Intelligence (Ward and Fischer, 2008; Shu et al., 2017; Chiesi et al., 2020). In this sense Mao and Liu (2016) provided evidence on the moderator role of social support into the relationships between Cultural intelligence and adaptation of Chinese international students.

### Limitations of the present study and implications for future research

The recruitment of participants through various social networks, while efficient in reaching a broad and diverse group of Chinese international students, introduces a potential selection bias. This approach might have limited the sample to students who are active on these platforms, potentially excluding those who do not use these social networks or have different usage patterns. Consequently, the findings may not fully represent the entire population of Chinese international students, affecting the generalizability of the results. Additionally, the response rate seems robust, but the exclusion of participants who did not complete 100% of the survey may lead to response bias. This criterion could overlook perspectives of students who chose not to complete the entire survey, possibly due to different experiences or views.

Furthermore, the cross-sectional design of the study limits the ability to establish causality between intrapreneurial self-capital, cultural intelligence, and flourishing. A longitudinal approach would be more suitable to understand how these relationships develop over time, particularly as students adapt to new cultural environments. Following this idea, some previous studies tested the predictive validity of Cultural Intelligence over time stating the importance of Motivational CQ as negative predictor of psychological problems during international adaptation (Ward et al., 2011).

The reliance on self-report measures for key constructs like Intrapreneurial Self-capital, Cultural Intelligence, and flourishing, despite using reliable scales, raises concerns about social desirability bias and individual differences in self-awareness. This may affect the accuracy of the responses.

The specific focus on Chinese international students also means that the findings might not be applicable to students from other cultural backgrounds or to Chinese students within their own country, limiting the study’s broader applicability. In this sense, some previous studies showed a relevant role of perceived cultural distance in the complex pattern of relationships between Cultural Intelligence and international students’ adjustment (Malay et al., 2023).

Moreover, the use of translated versions of scales, such as the Flourishing Scale Spanish Version, requires careful consideration. On the one hand, translation can sometimes alter the meaning of items, potentially impacting their interpretation and the validity of the results. On the other hand, some of the scales used in the present research have demonstrated their cross-cultural equivalence with different samples, included Chinese participants (Bücker et al., 2016; Schlägel and Sarstedt, 2016). These limitations highlight the need for cautious interpretation of the findings and suggest areas for improvement in future research.

Despite the recognized shortcomings, the study’s findings have important implications for the support and development programs for international students. Similar initiatives focused on the Intrapreneurial Self-capital training have been recently applied (McIlveen and Di Fabio, 2018). This kind of initiatives have proven efficacy in different contexts, as educative or professional fields (Azevedo and Shane, 2019; Zhang et al., 2022). Educational institutions and policymakers should consider tailoring their initiatives to enhance ISC and CQ, taking into account the different ways these factors interact based on gender. Furthermore, the nuanced roles of various CQ dimensions suggest that interventions should be multifaceted, focusing not only on knowledge and awareness but also on behavioral adaptation and motivational factors.

Future research could explore these relationships in different cultural contexts and among students from other backgrounds to understand the universality and specificity of these findings. Additionally, longitudinal studies could provide insights into how these relationships evolve over time, especially as students adjust to new cultural environments.

### Societal and policy implications

Firstly, the strong direct effect of Intrapreneurial Self-Capital on flourishing underscores the importance of educational policies that support the development of intrapreneurial skills such as self-initiative, resourcefulness, and resilience in international students. These skills are essential not only for academic success but also for psychological well-being in a cross-cultural setting. Educational institutions should integrate programs that foster these qualities, recognizing their role in enhancing the overall well-being of students.

Furthermore, the nuanced roles of different dimensions of Cultural Intelligence (Metacognitive, Cognitive, Motivational, and Behavioral) in this context suggest that educational and social programs need to be multifaceted. The substantial mediating effects of Motivational and Behavioral CQ, particularly among males, highlight the critical role of adaptive behavior and motivation in cultural environments. Educational programs should thus tailor their approaches to cultivate these specific aspects of Cultural Intelligence, recognizing their differential impact on student flourishing.

The study’s findings on the moderating effect of gender in the relationship between Intrapreneurial Self-Capital and Cultural Intelligence also have important implications. The pronounced gender differences, especially in Behavioral and Motivational CQ, suggest the need for gender-sensitive approaches in educational and social interventions. Policies and programs designed to facilitate the flourishing of international students should consider these gender-specific dynamics to be more effective.

In addition, the potential influence of other variables, such as personality traits and social support, on Cultural Intelligence and adaptation, underscores the complexity of these dynamics. Future research and policy development should consider these broader factors to fully understand and support the adaptation and flourishing of international students in cross-cultural environments.

## Conclusion

In conclusion, this study contributes to a deeper understanding of the factors contributing to the flourishing of Chinese international students. It highlights the critical role of intrapreneurial self-capital, the nuanced mediating roles of different dimensions of cultural intelligence, and the significant moderating effect of gender. These insights are invaluable in guiding efforts to support the well-being and adaptation of international students in multicultural settings. Overall, this study not only contributes to academic understanding but also provides practical insights for policymakers and educators in shaping supportive environments for international students. By acknowledging and addressing the complex interplay of intrapreneurial skills, cultural intelligence, and gender, policies can be more effectively tailored to enhance the well-being and success of this important student population.

## Data availability statement

The raw data supporting the conclusions of this article will be made available by the authors, without undue reservation.

## Ethics statement

The studies involving humans were approved by Ethical Committee Donghua University. The studies were conducted in accordance with the local legislation and institutional requirements. The participants provided their written informed consent to participate in this study.

## Author contributions

TD: Writing – original draft, Writing – review & editing. XL: Writing – original draft, Writing – review & editing.
